# Counter-Irritation with Tincture of Iodine in Neuralgia

**Published:** 1865-02

**Authors:** 


### COUNTER-IRRITATION WITH TINCTURE OF IODINE IN NEURALGIA.

In a clinical conference at the Children's Hospital in Paris, M. Bouchut relates several cases which illustrate the efficacy of this mode of counter-irritation in cases of neuralgia.

The first case related was that of a lady aged fifty, who for six months had been prevented by intercostal neuralgia from wearing stays. The painful region was painted over every day with tincture of iodine during a week, and at the expiration of that period a complete cure of was effected.

Another, and very corpulent lady, had for three years suffered from a stitch in the eighth intercostal space, in front of the short ribs. The same treatment was instituted, with perfect success.

A dyspeptic and hypochondriac subject affected with præcordial neuralgia, considered himself threatened with diseaes of the heart. Daily applications of tincture of iodine were prescribed, and the pain subsided; a relapse took place, and again the remedy, perseveringly resorted to with sufficient energy to cause desquamation, removed the neuralgia, which then disappeared altogether.

In some cases of obstinate intercostal neuralgia the patients imagine the symptom to be the indication of pulmonary tuberculosis. A lady, subject to bronchitis, had for twelve months suffered from a neuralgic pain of this description, and conceived herself to be consumptive; she resorted, by advice, to the Eaux Bonnes, but returned to Paris without having experienced any improvement. M. Bouchut applied the tincture of iodine, relieved the pain, and the much-dreaded thoracie disease was cured at the same time.

Neuralgia of the breast is to many women a cause of very great uneasiness, especially when the organ contains the small indurated lobules which M. Velpeau designates under the denominations of adenoma, or irritable tumor of the mamma. In these cases the patients are haunted by the fear of cancer, and M. Bouchut has had several opportunities of testing the efficacy of the tincture of iodine; applications daily repeated for eight or ten days have often succeeded in calming the pain, and in reassuring the patients.

In another case of a singular nature the neuralgia was accompanied by delusions of the sense of hearing, and the pain was so intense that the sufferer, tormented by hostile imaginary voices, and much alarmed by her symptoms, seemed bent on self-destruction. M. Bouchut removed the pain in a few days with the tincture of iodine, and this lady has since recovered from her melancholy mental condition.

M. Bouchut adduced several cases from which it appears that all varieties of neuralgia may be benefitted by the treatment he recommends.

A little girl was afflicted with supra-orbital neuralgia; and for three days in succession tincture of iodine was applied over the brow to a surface not exceeding the size of a sixpenny piece, and a cure was effected.

M. Bouchut further related two instances of hemicrania, with photophobia and vomiting, in which the symptoms yielded to the same remedy. He also brought forward a case of sciatica observed in a naval officer; the pain was chiefly apparent at the head of the fibula and near the tendo-Achilis.

Both regions were touched with the tincture, and the sciatica disappeared.

Blistering might possibly have proven equally beneficial; but the application of blisters is not always possible, and the tincture of iodine is a valuable and convenient substitute.

Thus, when M. Bouchut was attached as physician to the Hopital Sainte-Eugenie, he was consulted by a lady, residing at Charenton, for neuralgia in the occipital region. The patient was naturally desirous of preserving her hair, and instead of a blister, tincture of iodine was prescribed. The desired effect was produced after desquamation of the cuticle, and the hair was not injured.

As an instance of numerous and successive attacks of neuralgic pains, we may adduce the instance of a lady who, during her monthly period, wearing a crinoline and insufficient under-clothing, caught cold; the menses were checked, and retro-uterine hæmatocele followed. She was treated in an appropriate manner, and recovered her health; but pains supervened in the pelvis, between the shoulders, and in the intercostal spaces. The interscapular neuralgia had lasted eight months, when by M. Bouchut's advice, tincture of iodine was applied every day for a week; the treatment was very painful, but its results were perfectly satisfactory. Iliac neuralgia subsequently occurred; the same remedy was resorted to, but not with sufficient perseverance to secure permanent relief. Indeed, the method cannot be successful unless a predetermined action is induced, which hitherto can only be estimated by desquamation of the cuticle.

This is not a new mode of treatment. In pulmonary consumption, and in chronic pleurisy, the same procedure has been highly recommended for the relief of the pains which frequently accompany these affections. The method was introduced into practice by M. Blache, has since been very generally adopted, and is now acknowledged to be highly serviceable. This eminent practitioner, and also M. Van Holsbeck, in Belgium, and M. Magne, in Paris, have substituted with much benefit, tincture of iodine for the blisters formerly applied around the orbit, for the purpose of removing the photophobia which accompanies scrofulous ophthalmia, and interferes with the needful inspection of the eye-ball. This object is most satisfactorily attained by painting over the lids, forehead, and temples every second day with the tincture, and the tendency of blistered surfaces in hospital to become the seat of diphtheria is thus ingeniously neutralized.

